# Maternal adverse childhood experiences, postnatal depression, and early parenting behaviors: a study of microcoded mother–infant interaction in early infancy

**DOI:** 10.3389/frcha.2026.1772342

**Published:** 2026-04-01

**Authors:** Marcos Roberto Fanton, Mateus Mazzaferro, Karine Maria Porpino Viana, Danilo Micali, Vinicius Oliveira Santana, Ana C. C. Milani, Ivaldo Silva, Hugo Cogo-Moreira, Cristiane S. Duarte, Jonathan Posner, Andrea P. Jackowski

**Affiliations:** 1 Laboratory of Integrative Neurosciences (LiNC), Universidade Federal de São Paulo, São Paulo, Brazil; 2 Stanford Graduate School of Education, Stanford University, Stanford, CA, United States; 3 Department of Social Work, Child Welfare and Social Policy, Oslo Metropolitan University, Oslo, Norway; 4 Department of Health & Wellness Design, School of Public Health-Bloomington, Indiana University, Bloomington, IN, United States; 5 Department of Education, ICT and Learning, Østfold University College, Halden, Norway; 6 Department of Psychiatry, Columbia University Irving Medical Center, New York, NY, United States; 7 Department of Psychiatry, Duke University, Durham, NC, United States

**Keywords:** childhood adversities, depression, maternal behavior, mother-infant interaction, newborn

## Abstract

Human parenting behaviors (HPBs)—including gaze, motherese, affectionate touch, and positive affect—are essential for child development and can be precisely measured during the first weeks of life (2–8 weeks) through microcoding of videotaped interactions. This study examined whether maternal exposure to adverse childhood experiences (ACEs) affects HPBs and whether postnatal depressive symptoms mediate this relationship. The sample included 272 mother–infant dyads from São Paulo, Brazil (mean maternal age: 27.2 ± 5.2 years, 67% with monthly income < US$317; infants: 48.5% female, mean age: 33 ± 19 days). Maternal ACEs and depressive symptoms were self-reported during pregnancy and postnatally, respectively. HPBs were assessed using the Coding Interactive Behavior (CIB) manual. Results showed that maternal ACEs were significantly associated with higher postnatal depressive symptoms (*β* = 0.665, *p* < 0.001), but neither a direct nor indirect association with HPBs was observed. These findings suggest that ACE-related disruptions in parenting may not manifest during the early weeks of life but could emerge later as caregiving demands increase. This study is among the few to use microcoded interaction analysis at this early stage of life and highlights the need for continued research into the complex pathways linking ACEs, maternal mental health, and parenting behaviors over time.

## Background

The quality of mother–child interactions is strongly linked to a range of positive developmental outcomes, including improved language acquisition, cognitive abilities, motor skills, and social behaviors (1–3). Maternal behavior during these interactions can be characterized by a wide range of aspects, including human parenting behaviors (HPBs). HPBs, as a construct, have been supported as predictors of child development. For instance, they are associated with a child's ability to employ basic regulatory strategies, such as emotional self-regulation manifested through physical actions (e.g., thumb sucking) or verbal reassurances (e.g., telling oneself “It's okay”) (4). Notably, HPBs share similarities with the licking-and-grooming behaviors observed in other mammals, emphasizing their fundamental role in caregiving across species (1, 4).

HPB comprises four components: affectionate touch, motherese, maternal gaze, and maternal positive affect. Affectionate touch plays a key role in the development of emotional self-regulation and of the social brain (5–8), and it helps reduce withdrawal behavior in infants (9, 10). Similarly, the slower rhythm of “motherese” aids infants in speech segmentation, enabling them to identify and understand grammatical structures (11). This influence extends into later childhood, as research shows that children exposed to more maternal vocalizations tend to have higher verbal IQ scores (12). Moreover, the qualitative aspects of maternal speech, such as contingent responsiveness, are essential for effective dyadic communication and support socialization across different cultural contexts (13).

Regarding the gaze, mothers typically focus on their infants’ faces, especially their eyes, which enhances emotional bonding and social expressiveness (14). Infants are highly attuned to the direction of their mothers’ gaze, with even subtle shifts in horizontal gaze being significant (15). Direct eye contact has been linked to cognitive abilities in infants, such as face recognition (16), and is associated with higher quality of mother–child interaction at 6 and 12 months, as well as secure attachment (17).

Lastly, maintaining positive affect during interactions, such as smiling, can help infants adopt a positive approach when facing ambiguous situations in which they rely on their mother's cues to decide how to behave (18). Positive maternal affect is also associated with increased sensitivity and better synchrony between mother and infant (19). Furthermore, the social smile, an important developmental milestone, often emerges as a contingent response to the mother's smile (20, 21). Thus, the consistency of positive maternal affect, alongside other maternal characteristics, is crucial for fostering infants’ social and emotional communication abilities (22).

Despite the positive outcomes associated with HPB and its components, adverse childhood experiences (ACEs) can profoundly impact a mother's relationship with her child. ACEs encompass various forms of child maltreatment, including physical, emotional, and sexual abuse, as well as physical and emotional neglect. They also include aspects of family dysfunction, such as parental substance abuse, mental illness, domestic violence, and criminal behavior (23).

The link between ACEs and the quality of mother–child interactions is marked by a small yet significant negative pooled correlation, as found in a meta-analysis conducted by Savage et al. (24). A more recent review found that 90% of the studies analyzed reported a negative association between the number of maternal ACEs and maternal positive behaviors (25). In a rare study that examined the relation between ACEs and specific HPBs, it was found that mothers who were exposed to more ACEs showed less positive affect, motherese, and gaze directed to their three-month-old infants (26).

Moreover, ACEs have profound and lasting effects on individuals’ cognitive, social, and emotional development, often impairing their functioning across the lifespan (27). Those exposed to ACEs are more likely to experience a range of physical, social, and psychological challenges, with depression being particularly prevalent—individuals with ACEs are twice as likely to develop depression, regardless of sex or age at exposure (28). Additionally, ACEs are associated with perinatal depressive symptoms, with small effect size reported for the prenatal period (*d* = 0.39) and medium effect size for the postnatal period (*d* = 0.47) (29).

Depression further disrupts maternal behaviors and interactions with the child, often resulting in disengagement or intrusive behaviors (30, 31). Furthermore, a systematic review showed that depression may mediate the association between ACEs and the quality of mother–child interactions (32). This effect can be observed even during pregnancy, with ACEs altering maternal–fetal attachment through depression (33).

However, few studies have investigated the influence of ACEs on maternal behavior, here characterized as HPBs, during the very early stages of an infant's life (24, 25, 32). Up to approximately two months of age, infants exhibit limited social orientation, which changes thereafter with the onset of the two-month shift (34). Thus, studying this early period provides clearer insight into how ACEs influence maternal behavior in a context where the mother's responses are less shaped by the infant's increasing social engagement. Despite global aspects of maternal behavior being widely covered in the existing studies, fine-grained, microanalytical methods are underrepresented and warrant further investigation.

This study examined the association between maternal ACEs and HPBs, including gazing at an infant's face, positive affect (smiling), motherese, and affectionate touch. Additionally, we investigated whether maternal depressive symptoms during the early weeks of life mediated this association. We hypothesized that mothers with higher ACE scores would demonstrate less frequent HPBs (H1) and that maternal postnatal depressive symptoms would mediate this association (H1a).

## Methods

### Sample

This study is part of the Healthy MiNDS cohort, a longitudinal project designed to evaluate how maternal ACEs shape infant neurodevelopment and the underlying genetic and hormonal pathways. The cohort also tracks key domains of early development, including cognitive, language, socioemotional, and motor functioning (35).

Women were eligible to participate if they: (1) resided in socially vulnerable, low-resource neighborhoods in the municipalities of Guarulhos or São Paulo, Brazil; (2) received prenatal care through the Brazilian public health system (Sistema Único de Saúde—SUS); (3) were between 18 and 38 years of age; (4) were in the late second to third trimester of pregnancy, specifically between 25 and 39 gestational weeks; and (5) demonstrated adequate literacy to comprehend the study information and voluntarily signed written informed consent.

Pregnant women were excluded if they met any of the following conditions: (1) classification as a high-risk pregnancy; (2) BMI (body mass index) > 30; (3) diagnosis of a severe psychiatric disorder, such as schizophrenia, chronic delusional disorder, bipolar disorder, obsessive–compulsive disorder, dementia, or active suicidal ideation; (4) history of neurological compromise, including traumatic brain injury, epilepsy requiring treatment, or previous neurosurgical procedures; (5) uncontrolled medical illnesses requiring intensive clinical management; (6) current use of illicit substances (with the exception of cannabis); or (7) laboratory-confirmed infections classified within the TORCH group (toxoplasmosis, syphilis/other, rubella, cytomegalovirus, or herpes).

Newborns were enrolled immediately after delivery when their mothers reaffirmed their willingness to continue in the cohort. Infants were excluded if they: (1) were born preterm (<37 weeks of gestation); (2) had a birth weight below 2.5 kg; (3) received an APGAR score lower than 7 at five minutes of life; (4) required admission to a neonatal intensive care unit; or (5) presented conditions such as kernicterus or diagnosed inborn errors of metabolism.

### Measures

#### Maternal ACEs

The CDC-Kaiser ACE Questionnaire was administered during pregnancy and consists of 10 dichotomous (yes/no) items assessing exposure before age 18 to abuse (emotional, physical, and sexual), neglect (emotional and physical), and household dysfunction (including domestic violence, parental separation or divorce, substance abuse, mental illness, and incarceration of a household member). The total ACE score is calculated as the sum of endorsed categories (ranging from 0 to 10), reflecting cumulative exposure to early-life adversity (23, 36).

#### Depressive symptoms

The Patient Health Questionnaire-9 (PHQ-9) was applied during recruitment and at any time prior to the date of the mother–infant interaction recording (2–8 weeks postpartum). The PHQ-9 comprises nine items that assess symptoms of major depressive disorder as defined in the DSM-IV, measuring their frequency over the preceding two weeks. Each item is rated on a Likert scale. The PHQ-9 allows individuals to be classified in different levels of depression according to the total score (range: 0–27): no or minimal (0–4); mild (5–9); moderate (10–14); moderately severe (15–19), or severe (20–27). In the present study, only the total score was used in the analyses (37, 38).

#### Maternal race, educational level, and socioeconomic status

Mothers self-reported their race or ethnicity and their level of educational attainment. Socioeconomic status (SES) was assessed using the Associação Brasileira de Empresas de Pesquisa (ABEP) scale, which evaluates household conditions, including the number of appliances and electronic devices, cars, presence of a housekeeper, access to clean piped water, the paving condition of the street (paved or unpaved), and the educational level and monthly income of the family's primary earner. Each item receives a score, and the total score is converted into an SES classification system (A1, B1, B2, C1, C2, and DE), with DE indicating the lowest socioeconomic level (39). In the present analysis, the raw total score was used to retain the full variability of socioeconomic conditions and avoid the loss of information inherent in categorical classification.

#### Videotaping and microcoding

A total of 344 mother–infant dyads attended the laboratory visit for videotaping. Of these, 59 could not be recorded due to the infant's behavioral state (fussiness, crying, or sleeping). Among the recorded videos, seven were lost due to technical problems and six were excluded because of improper camera framing, resulting in 272 analyzable videos.

When infants were 2–8 weeks old, mothers participated in a standardized five-minute interaction task. Seated with their infants on their laps and facing each other, they were instructed to interact naturally, “as they usually do,” without the use of toys, music, or other objects. The camera was positioned to capture the mother's upper body, including both faces, and the experimenter monitored the recording remotely.

Recordings were attempted only when the infant was in a quiet, alert state prior to the start of the session. If the infant was already fussy, crying, or asleep before filming began—potentially due to extraneous factors such as the laboratory environment or prior study procedures—the dyad was excluded from recording to ensure that the observed interaction reflected the mother's typical behavior and the dyadic process rather than preexisting infant distress. Furthermore, if mothers began breastfeeding during the videotaping procedure, the recording was interrupted and restarted afterward. This ensured a standardized, non-feeding context for the interaction, although breastfeeding could be used beforehand to calm a distressed infant.

Microcoding was conducted from seconds 60 to 240 to allow adaptation during the first minute. Coders annotated the onset and offset of each behavior using the Behavioral Observation Research Interactive Software (BORIS, version 7.13.8) (40). The ethogram followed the Coding Interactive Behavior Manual–Newborn (CIB-Newborn)^1^ and included maternal gaze to the infant's face, positive affect (smiling), affectionate touch, and motherese vocalization.

Smiling was identified through activation of the zygomaticus major muscle (AU12), with or without activation of the *orbicularis oculi* (AU6), characteristic of a Duchenne smile (41, 42). Affectionate touch included massaging, caressing, kissing, and sniffing. Motherese was defined by its higher pitch, exaggerated prosody, slowed tempo, and simplified linguistic structure (43).

After coding, BORIS automatically computed the proportion of time each behavior occurred. Two trained coders (MRF and MM), blinded to maternal ACEs and depressive symptoms, performed all coding. Training was completed with the CIB team until ≥85% agreement was reached, and inter-rater reliability for the study was high (Cohen's *κ* = 0.86).

### Statistical analysis

Exploratory correlation analyses were first conducted among all variables to examine their relationships before testing the main hypothesis. Mediation modeling under the counterfactual approach (44, 45) was used to test the hypothesis that ACEs would impact maternal behaviors directly and indirectly via maternal depression. The counterfactual approach is more flexible than the standard statistical approach, allowing for interaction between the exposure and the mediator (46).

HPB is a latent variable underlying four observed behaviors: gazing at the infant's face, positive affect, motherese, and affectionate touch. Using a latent variable as a continuous outcome allowed the model to reduce the false discovery rate due to multiple-outcome comparison in the context of a mediation model, and to create a more reliable measure by grouping and evaluating behaviors with the same cause. The latent variable, HPB, was tested separately before being integrated into the mediation model using confirmatory factor analysis (CFA). Maximum likelihood estimation was used and the cutoffs for evaluating the HPB model's fit were established using a dynamic model fit approach (47, 48).

Because of missing data (which is commonly observed in longitudinal studies), two models were executed: one with listwise, in which bootstrapped bias-corrected confidence intervals (CIs) were generated, and another with multiple imputations, assuming a missing-at-random mechanism, in which regular confidence intervals were generated. This sensitivity analysis was conducted to evaluate the impact of missingness on the significance, direction, and magnitude of the estimate.

Twenty datasets were imputed after the convergence of the Bayesian iterative sequence, and simulation studies showed that five imputed datasets were often sufficient (49, 50). Subsequently, the datasets were analyzed using the frequentist estimation method (maximum-likelihood, given that the outcomes for the mediation model were continuous), the parameter estimates were obtained as the average over the different analyses, and the standard error for the parameter estimates was computed via the *Schafer 6* formula (50). The imputation method was the covariance model, which uses an unrestricted mean.

All analyses were conducted using Mplus version 8.0. As standardized effects are not available when performing multiple imputations, the reported effects are presented as unstandardized linear regression coefficients.

### Ethics

This study was approved both in Brazil by the Comitê de Etica em Pesquisa (CONEP, process number 78018417.2.1001.5505) and at the partner institutions in the United States, namely: New York State Psychiatric Institute [NYSPI Institutional Review Board (IRB), protocol number 7927] and Duke Health (IRB protocol number 00110664). Textual and image data were securely stored in institutional servers.

## Results

The mean maternal age was 27 ± 5.2 years (range: 18–38 years) and had 3–9 years of formal education (mean: 7 ± 1 years). Mean maternal age was 27 ± 5.2 years (range: 18–38 years), and participants had between 3 and 9 years of formal education (mean: 7 ± 1 years). The majority of the sample (approximately 67%) fell within the two lowest socioeconomic status categories, corresponding to a monthly income below US$317. Infants were 48.5% female, with a mean age of 33 ± 19 days (range: 14–59 days). Nearly half of the women (40%) were single mothers, 20% reported a history of miscarriage, and 50% were primiparous. Additional sociodemographic characteristics are provided in Table 1.

**Table 1:** Sample description.

Variable	*N*	CIB available (*n* = 272)	CIB not available (*n* = 355)	Statistic test
Maternal age	614			
Mean ± SD Range		27.2 ± 5.2 18–38	25.9 ± 5.3 18–38	F_1,612_ = 10.05, *p* = 0.01^a^
Maternal self-reported race	613			
Asian		20 (7%)	38 (11.1%)	*χ*^2^_4_ = 4.65, *p* = 0.32^b^
Black		45 (16%)	64 (18.8%)	
Indigenous		1 (1%)	0 (0.0%)	
Multiracial		109 (40%)	125 (36.7%)	
White		97 (35%)	114 (33.4%)	
Maternal socioeconomic status	534			
A-B1		6 (2.2%)	2 (0,8%)	χ^2^_1_ = 2.64, *p* = 0.62^b^
B2		25 (9.3%)	24 (9.0%)	
C1		53 (19.8%)	49 (18.6%)	
C2		98 (36.6%)	96 (36.1%)	
D-E		86 (32.1%)	95 (35.7%)	
Maternal marital status	610			
Single		106 (39.0%)	137 (40.5%)	χ^2^_2_ = 4.60, *p* = 0.10^b^
Married		157 (57.7%)	198 (58.6%)	
Divorced/Separated		9 (3.3%)	3 (0.9%)	
Widowed		0 (0%)	0 (0%)	
Number of children prior to the birth of the enrolled infant	601			
0		126 (47.2%)	169 (50.6%)	χ^2^_4_ = 5.23, *p* = 0.26^b^
1		93 (34.8%)	94 (28.1%)	
2		30 (11.2%)	48 (14.4%)	
3		9 (3.4%)	16 (4.8%)	
4 or more		9 (3.4%)	7 (2.1%)	
Maternal history of miscarriage	598			
0		195 (73.6%)	169 (50.6%)	χ^2^_1_ = 1.54, *p* = 0.21^b^
1		60 (22.6%)	94 (28.1%)	
2		9 (3.4%)	48 (14.4%)	
3		0 (0%)	16 (4.8%)	
4		1 (0.4%)	7 (2.1%)	
Infant biological sex	545			
Female		132 (48.5%)	147 (53.8%)	χ^2^_1_ = 1.54, *p* = 0.21^b^
Male		140 (51.5%)	126 (46.2%)	
Maternal ACEs	604			
Mean ± SD Range		3.5 ± 2.4 0–10	3.4 ± 2.3 0–10	F_1,602_ = 0.45, *p* = 0.50^a^
Maternal Behaviors Relative Frequency [Median (Range)]	272			
Positive affect		14% (0%—97%)	–	–
Motherese		57% (0%—100%)	–	
Gaze at the infant's face		92% (0%—100%)	–	
Affectionate touch		9% (0%—58%)	–	

N is the number of non-missing value. ACEs, adverse childhood experiences; SD, standard deviation; CIB, Coding Interactive Behavior Manual–Newborn. Socioeconomic classification was based on criteria established by the Associação Brasileira de Empresas de Pesquisa (ABEP). The estimated mean income for each stratum were as follows: A (US$ 4,629.40), B1 (US$ 2,043.29), B2 (US$ 1,022.00), C1 (US$ 558.96), C2 (US$ 316,77), and D/E (<US$ 130.40). Using the exchange rate of US$ 1 = R$ 5.52.

^a^ Wilcoxon.

^b^ Pearson.

The mean ACE score was 3.5 ± 2.4 and 75% of the sample reported experiencing two or more types of ACEs (Supplementary Figure S1). Parental divorce was the most frequently reported ACE (18%), whereas physical neglect and domestic violence were the least reported (5% each) (Supplementary Figure S2). Parental divorce was not only the most frequently reported ACE but also the one that was most often reported in isolation (absolute frequency = 45), meaning that participants reported no other ACE besides parental divorce. In addition, eight participants reported all types of ACEs, and the most common co-occurrence of two ACEs was between parental divorce and parental psychopathology, as reported by 12 participants (Supplementary Figure S3).

The mean score of depressive symptoms was 6.7 ± 4.9. Depression severity in the sample was distributed as follows: 39% of the participants had no or minimal depression, 32% had mild, 18% had moderate, and 10% had moderately severe or severe depression.

As shown in Table 1, the most prevalent behavior observed among mothers was gazing at their infants’ faces, with median engagement occurring during 92% of the interaction time. In contrast, affectionate touch was the least frequent behavior, with a median engagement of only 9% of the interaction time.

No statistically significant differences were observed between cohort participants with and without CIB data in maternal ACE scores or other sociodemographic variables, except for maternal age (F(1,612) = 10.05, *p* = 0.011). However, the magnitude of this difference was small (mean difference = 1.3 years; d ≈ 0.25), with substantial overlap in age distributions, indicating no meaningful difference between the two groups (Table 1).

### HPB latent model

**Figure 1:**
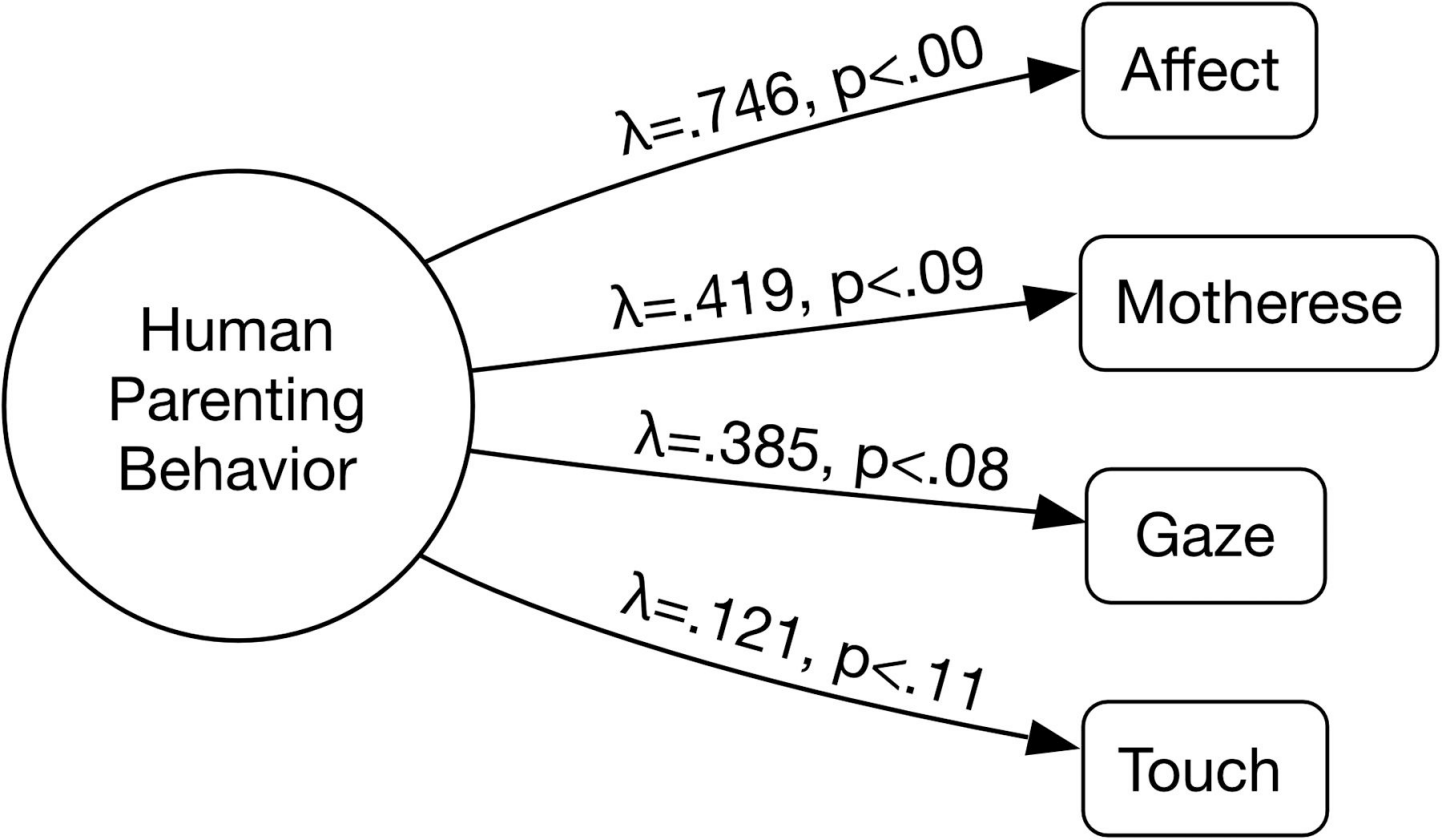
Confirmatory factor analysis (CFA) model. Affect=maternal expression of facial positive affect; Mothrese=maternal expression of motherese-like vocalizations directed to the infant; Gaze=maternal gaze directed to the infant's face; Touch=maternal affectionate touch.

The CFA showed that the HPB model, represented by the four observed behaviors, had a good fit [*χ*^2^(2) = 0.968, *p* = 0.6164, RMSEA = 0.000 (90% CI: 0.000–0.097, *p* = 0.775), CFI = 1.000, TLI = 1.000, and SRMR = 0.014]. Figure 1 presents a diagram of the HPB model with standardized factor loadings and their respective *p*-values.

The behavior with the highest loading was positive affect (*λ* = 0.746, *p* < 0.001), whereas affectionate touch showed the lowest loading (*λ* = 0.121, *p* < 0.114). Motherese and gazing at the infant's face had intermediate factor loadings (*λ* = 0.419, *p* = 0.09 and *λ* = 0.385, *p* = 0.084, respectively).

### Correlation analysis

Supplementary Table S1 presents a correlation matrix among the variables included in the main analysis. The matrix provides important preliminary insights that support the mediation analysis and help verify key assumptions. First, the weak-to-moderate correlations among the variables suggest that multicollinearity is not a concern, indicating that each predictor contributes unique variance to the model. Additionally, the positive correlation between ACEs and maternal depression (*r* = 0.36) supports the plausibility of depression as a potential mediator. The absence of a clear correlation between ACEs and HPB (*r* = −0.07), as well as between maternal depression and HPB (*r* = −0.11), suggests that any indirect effect would not be apparent at the bivariate level, reinforcing the need for a mediation model to assess whether maternal depression functions as a mechanism linking ACEs to HPB.

### Hypothesis testing

Table 2 presents the effects of the maternal ACEs on the mediator (depressive symptoms) and outcome (HPB) under listwise (top) and multiple imputation (bottom). A statistically significant association was observed between maternal ACEs and maternal depressive symptoms (*β* = 0.665, *p* < 0.001), both after listwise and multiple imputations. The mother's age and depressive symptoms were also associated (*β* = –0.166, *p* = 0.0051), but only listwise. None of the paths reaching HPB were statistically significant (highlighted in italics).

**Table 2:** Effect of ACEs on maternal depression and HPB.

Covariates	Effect on maternal depressive symptoms				Effect on HPB			
	Estimate	LBCI	UBCI	*P*-value	Estimate	LBCI	UBCI	*P*-value
Listwise (*n* = 173)								
1) Mother's age	**−0**.**166**	**−0**.**287**	**−0**.**056**	**0**.**005**	−0.005	−0.045	0.048	0.839
2) Education	−0.117	−0.876	0.450	0.725	0.148	−0.077	0.366	0.194
3) SES	−0.065	−0.181	0.031	0.219	−0.004	−0.041	0.034	0.822
4) Infant sex	0.690	−0.621	1.991	0.311	0.321	−0.116	0.799	0.165
5) Infant age	0.006	−0.074	0.084	0.881	0.020	−0.004	0.042	0.094
6) ACEs	**0**.**665**	**0**.**351**	**0**.**956**	**<0**.**001**	−*0*.*094*	−*0*.*290*	*0*.*106*	*0*.*362*
7) Depressive symptoms	–	–	–	–	0.000	−0.089	0.082	0.994
Interaction (6 and 7)	–	–	–	–	0.001	−0.017	0.021	0.894
Multiple imputation (*n* = 614)								
1) Mother's age	−0.106	−0.217	0.005	0.062	0.004	−0.031	0.038	0.838
2) Education	−0.213	−0.878	0.451	0.529	−0.001	−0.149	0.147	0.989
3) SES	−0.095	−0.196	0.006	0.066	0.006	−0.026	0.037	0.732
4) Infant sex	0.018	−1.149	1.186	0.976	0.053	−0.355	0.461	0.799
5) Infant age	−0.002	−0.072	0.067	0.946	0.002	−0.066	0.010	0.614
6) ACEs	**0**.**684**	**0**.**462**	**0**.**906**	**<0**.**001**	−*0*.*068*	−*0*.*174*	*0*.*038*	*0*.*208*
7) Depressive symptoms	–	–	–	–	0.023	−0.068	0.113	0.624
Interaction (6 and 7)	–	–	–	–	−0.002	−0.013	0.009	0.759

Significant variables are indicated in bold.

Table 3 lists the four counterfactual-based effects and their 95% confidence intervals. None of the counterfactual-derived effects (direct and indirect) were statistically significant as all 95% confidence intervals crossed zero under both listwise and multiple imputation, showing convergent results.

**Table 3:** Counterfactual-based effects.

Effect	Estimate	LBBCI	UBBCI	UBOCI
Listwise (*n* = 173)				
Total Natural Indirect Effect	0.001	−0.053	0.051	–
Pure Natural Direct Effect	−0.082	−0.202	0.022	–
Total Effect	−0.082	−0.182	0.029	–
Pure Natural Indirect Effect	0.000	−0.060	0.060	–
Total Natural Direct Effect	−0.081	−0.202	0.025	–
Multiple imputation (*n* = 614)				
Total Natural Indirect Effect	0.015	−0.041	–	0.072
Pure Natural Direct Effect	−0.090	−0.193	–	0.013
Total Effect	−0.075	−0.166	–	0.017
Pure Natural Indirect Effect	0.017	−0.045	–	0.078
Total Natural Direct Effect	−0.091	−0.198	–	0.016

LBBCI, 95% lower bound bias-corrected confidence interval; UBBCI, 95% upper bound bias-corrected confidence interval; LBOCI, 95% lower bound observed confidence interval; UBOCI, 95% upper bound observed confidence interval.

## Discussion

This study aimed to investigate the association between maternal ACEs and HPBs during the first weeks of life (2–8 weeks) and to explore the mediating role of maternal postnatal depression. We hypothesized that higher ACE scores would be associated with less frequent HPBs (gaze, positive affect, touch, and vocalization) and that maternal depressive symptoms would mediate this association. Contrary to current literature, our main hypotheses were not supported, as no statistically significant direct or indirect effects of ACEs on HPBs were found. Additionally, no relationship was found between maternal postnatal depressive symptoms and HPBs. Notably, maternal depressive symptoms were significantly associated with maternal ACEs, corroborating prior findings that highlight ACEs as a robust predictor of maternal mental health problems (28, 51).

Our study is among the few to evaluate maternal behaviors during interactions with infants younger than three months old in relation to maternal ACEs. Although summarizing prior findings is challenging due to methodological heterogeneity, most studies report an effect of maternal ACEs on mother–child interactions, either directly or indirectly through depression, post-traumatic stress disorder (PTSD), stress, or neurophysiological functioning (24, 25, 32).

The lack of statistically significant findings regarding the direct association between ACEs and HPBs in our study warrants further exploration. As stated in the Introduction, Riva Crugnola et al. (26) found associations between maternal ACEs and specific nurturing behaviors directed toward 3-month-old infants. It is well-established that around two months of age, infants undergo a qualitative shift in behavior, becoming more socially oriented and capable of reciprocal exchange (34). This developmental transition introduces new interactive demands that require mothers to engage in increasingly complex, contingent, and mental-state-oriented behaviors. Therefore, it is possible that the effects of maternal ACEs on HPBs become more pronounced or more easily detectable only after this shift, when the interaction presents new features requiring greater maternal social-cognitive flexibility.

Importantly, the fact that Riva Crugnola et al. (26) detected ACE effects as early as three months using a different methodological approach also raises the possibility that our null findings may partly reflect the limitations of our specific protocol. As discussed earlier, our short, unstructured, object-free laboratory interaction may not have been optimally sensitive to capture the subtle influences of ACEs on maternal behavior, particularly in a low-distraction setting where mothers can more easily marshal their attentional resources. Future studies employing longer, more varied, or more challenging interaction paradigms may be better equipped to detect these effects earlier in development.

It is also important to note that Riva Crugnola et al. (26) used group comparisons in their analysis, whereas the present study treated participants as a continuous variable ranging from no reported ACEs to the maximum score. This analytic strategy was adopted because continuous modeling captures finer variations in exposure and may provide more nuanced insights (52–54). Measurement tools used to assess HPBs and ACEs also differ across our prior studies. In the present study, individual discrete behaviors were analyzed separately (maternal gaze, affect, vocalization, and touch), whereas Riva Crugnola et al. (26) grouped behaviors into broader categories encompassing multiple sets of discrete actions. For example, their “negative engagement” category included behaviors such as negative affect, withdrawal, intrusiveness, and hostility, while “social positive engagement” encompassed positive facial expressions, motherese, and social play. Additionally, the instrument used to assess ACEs in their study did not capture all types of adversities examined here. Notably, it excluded parental divorce, which was the most frequently reported adversity in our sample.

The lack of association between depressive symptoms and maternal behavior can be interpreted through several perspectives. A meta-analysis identified three key moderators: socioeconomic status, child's age, and observation methodology (31). Our sample comprised primarily low-SES mothers with young infants, conditions under which stronger associations are tipically observed. However, the relatively low prevalence of high depressive symptom levels in our sample may have limited the ability to detect such effects. Methodologically, although observations were conducted in a laboratory setting, the interaction task was unstructured, a format that tends to yield weaker associations than structured paradigms. Similar results have been reported in studies that employed a comparable methodology (55).

It is also important to acknowledge the limitations inherent in our observational approach. Microanalysis of a three-minute, unstructured mother-infant interaction in a laboratory setting provides a valuable but narrow window into a complex and dynamic relationship. Such a brief observation may not fully capture the range and quality of behaviors that occur in the naturalistic home environment (56, 57). The unstructured task, although intended to elicit natural behavior, is susceptible to the participants’ transient states, such as infant fussiness, which led to the exclusion of a subset of dyads and may have biased our sample toward more regulated interactions. This trade-off between ecological validity and experimental control was deliberate, prioritizing the high degree of standardization required for fine-grained microcoding of discrete behaviors like gaze and touch (58). The controlled setting optimized audio and video quality, which is particularly critical for reliability in our low-income sample, where home environments often lack the quiet, uninterrupted space required for high-quality recordings. Furthermore, the three-minute duration was pragmatically chosen to balance methodological rigor with the feasibility of coding a large cohort and is arguably adequate for capturing core HPBs during the neonatal period, when infant behavioral repertoires are less varied. Nevertheless, these limitations constrain the generalizability of our findings. Future research should complement this approach with longer, more varied observations, including home-based assessments, to provide a more ecologically valid and comprehensive understanding of how maternal ACEs and depression shape caregiving behaviors over time, as planned in subsequent phases of this longitudinal study.

Beyond the influence of observational duration and structure, it is also important to consider how the specific laboratory context may have shaped the absolute levels of the behaviors we measured. The high frequency of maternal gaze directed at the infant's face (median = 92%) likely reflects, in part, the structured nature of the setting, in which mothers and infants were seated face-to-face without toys or other distracting objects. This paradigm, while standardized and widely used in the field (21, 59), explicitly invites maternal attention toward the infant and may therefore elevate engagement behaviors compared with naturalistic home environments. However, this does not appear to compromise the validity of our findings. First, substantial interindividual variability was still observed (range: 0%–100%), indicating that the measure captures genuine differences in maternal engagement rather than a mere ceiling effect. Second, from an ethological perspective, high levels of gaze during face-to-face interactions in early infancy are considered a normative and species-typical behaviors essential for bonding and social development (4, 14). Thus, although the procedure may have optimized conditions for observing gaze, it likely reflects the upper bound of a maternal capacity for focused social attention. This trade-off between ecological validity and experimental control is acknowledged as a limitation, and future studies employing home-based observations will be valuable for complementing these laboratory findings with more naturalistic data.

An alternative interpretation worth considering—although not testable in the present study—is that individual or social resilience may buffer the potential negative impacts of ACEs and depression on early maternal behaviors. Notably, the negative effects of ACEs on mental health during pregnancy are more pronounced among women with lower resilience (60). Because resilience was not assessed in our sample, it is not possible to determine whether higher levels of resilience among our participants may have attenuated the expected association between ACEs and HPBs. This highlights an important direction for future research: studies should include validated measures of resilience to examine whether it moderates the pathways linking maternal adversity, mental health, and early caregiving behaviors. Understanding the role of resilience—at both the individual and social levels—may help explain why some mothers sustain positive parenting behaviors despite exposure to ACEs, whereas others do not.

Furthermore, research has shown that mothers who have effectively resolved potentially traumatic childhood experiences are better able to guide emotionally supportive conversations with their children (61). Trauma resolution refers to the process through which individuals integrate traumatic experiences into their internal narratives in a manner that enables present-oriented functioning. In contrast, unresolved trauma may manifest as distorted cognitions, such as persistent self-blame related to the traumatic event. These findings suggest that trauma resolution may play a crucial role in mitigating the negative outcomes associated with ACEs. In this context, the absence of a link between ACEs and maternal behaviors, despite significant associations with depressive symptoms, underscores the complex interplay of factors influencing parenting.

The significant negative association between maternal age and depression is consistent with existing literature. Prior research indicates that factors such as stress and lack of support (62), socioeconomic challenges (63), and increased expectations and role strain (64) disproportionately affect younger mothers, making them more vulnerable to depression. However, in our study, this finding should be interpreted cautiously, as the effect did not persist after multiple imputation. This suggests that the result may have been influenced by bias in the complete-case data used in the listwise analysis.

Socioeconomic inequality is a defining feature of the Brazilian context and plays a central role in shaping exposure to ACEs. As a low- and middle-income country (LMIC), Brazil reports high levels of early adversity, with population-based studies indicating that most individuals have experienced at least one ACE, exceeding rates observed in high-income countries (65–68). National data from the Brazilian Institute of Geography and Statistics (IBGE) (69) indicate that the majority of the population identifies as Black or multiracial, groups disproportionately affected by socioeconomic disadvantage. In this context, structural inequities are closely linked to cumulative exposure to adversity, and Black and multiracial children are more likely to report multiple ACEs than their White peers. Because our sample largely comprised families from low socioeconomic backgrounds, broader contextual stressors may have influenced caregiving behaviors such as HPB beyond the specific contribution of maternal ACE exposure. To address this potential confounding factor, socioeconomic status was statistically controlled in all analyses. Nevertheless, socioeconomic disadvantage may shape parenting practices through mechanisms extending beyond individual-level adversity, including chronic stress, limited access to resources, and structural constraints. Accordingly, our findings should be interpreted within this broader sociostructural context.

Overall, although our study did not support the hypothesized direct or indirect effects of ACEs on HPBs during the first weeks of life, it contributes to a growing body of research on this complex topic. These non-significant findings highlight the need for further investigation incorporating additional moderating and mediating factors, such as parental stress and trauma resolution. They also underscore the importance of adopting non-deterministic perspectives when considering mothers exposed to ACEs.

The strengths of this study include the assessment of maternal behavior during the very early stages of the infant life and its longitudinal design. However, focusing solely on the early postnatal period provides only a snapshot of mother-infant interactions, and, as discussed earlier, the effects of maternal ACEs may become more pronounced at later developmental stages. Additionally, the study relied on retrospective self-reported ACE measures, which are susceptible to recall biases. Both ACEs and depressive symptoms were assessed via maternal self-report, raising the possibility of common method variance. However, this concern is mitigated by the temporal separation between ACE assessment (during pregnancy) and depressive symptom assessment (postnatally), as well as by the use of objective, independent microcoding for the primary outcome (HPB), which substantially reduces the risk of method bias inflating observed associations (70).

It is also important to consider the duration and context of our observational measure. The five-minute face-to-face interaction, although brief, follows the standardized and validated protocol of the Coding Interactive Behavior (CIB) Manual—Newborn (see text footnote 1), which was specifically designed for infants in the first weeks of life. At this age, infants have limited attentional capacity, and longer interactions would likely exceed their ability to remain engaged, increasing fatigue and data loss. This duration is consistent with a substantial body of research employing well-established instruments for this developmental period (26, 71). Nevertheless, we acknowledge that a single five-minute laboratory observation cannot fully capture the complexity and variability of everyday mother-infant interactions at home. Mothers may also regulate their behavior more consciously in this context, potentially introducing social desirability bias. Importantly, such limitations would be expected to attenuate rather than inflate associations between risk factors and maternal behavior, rendering our null findings more conservative. Future research should ideally include multiple and longer observations across different contexts to provide a more ecologically valid and comprehensive understanding. Our ongoing longitudinal study addresses this limitation by incorporating home-based observations at later time points (6, 14, 18, and 24 months), which will allow for comparisons across settings and developmental stages.

Future research should also examine additional stages of infant and child development to determine whether the effects of ACEs on HPB emerge over time. Including measures of additional mediating factors, such as parenting stress, and moderating factors, such as resilience and trauma resolution, may provide a more nuanced understanding of the pathways linking maternal adversity to caregiving behaviors. Qualitative studies treating the dyad as the unit of analysis may further deepen understanding of how maternal ACEs influence interaction quality.

## Data Availability

The datasets used in this study are partially available through the NIMH Data Archive (NDA; https://nda.nih.gov) under collection C3811. Additional datasets cannot be made publicly available due to ethical and data protection requirements, as they may contain identifiable participant information. De-identified or derived data supporting the findings of this study may be made available by the corresponding author upon reasonable request.

## Footnotes

^1^Feldman R. *Coding Interactive Behavior Manual—Newborn* (1998).
