# Supplementary material for: Maternal adverse childhood experiences, postnatal depression, and early parenting behaviors: a study of microcoded mother–infant interaction in early infancy
Source: Front Child Adolesc Psychiatry. 2026 Apr 1;5:1772342. doi: 10.3389/frcha.2026.1772342 (PMC13079283; doi:10.3389/frcha.2026.1772342)
Supplement: Supplementary file 1 [file Presentation1.pdf]

Maternal Adverse Childhood Experiences, Postnatal Depression, and Early Parenting Behaviors: A Study of Microcoded Mother–Infant Interaction in Early Infancy

Marcos Roberto Fanton, Mateus Mazzaferro, Karine Maria Porpino Viana, Danilo Micali, Vinicius Oliveira Santana, Ana C. C. Milani, Ivaldo Silva, Hugo Cogo-Moreira, Cristiane S. Duarte, Jonathan Posner, Andrea P. Jackowski

SUPPLEMENTARY MATERIAL

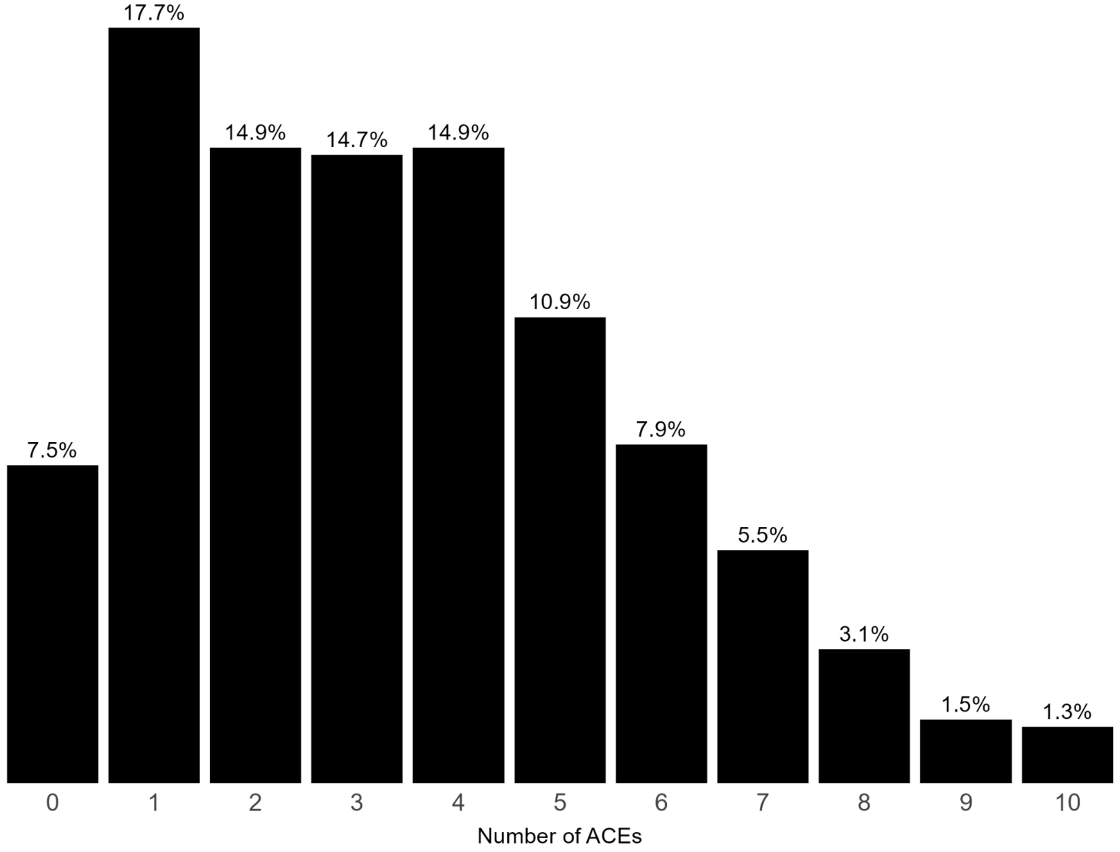

Figure S1. Maternal ACEs Total Score Proportions. ACEs: Adverse Childhood Experiences.

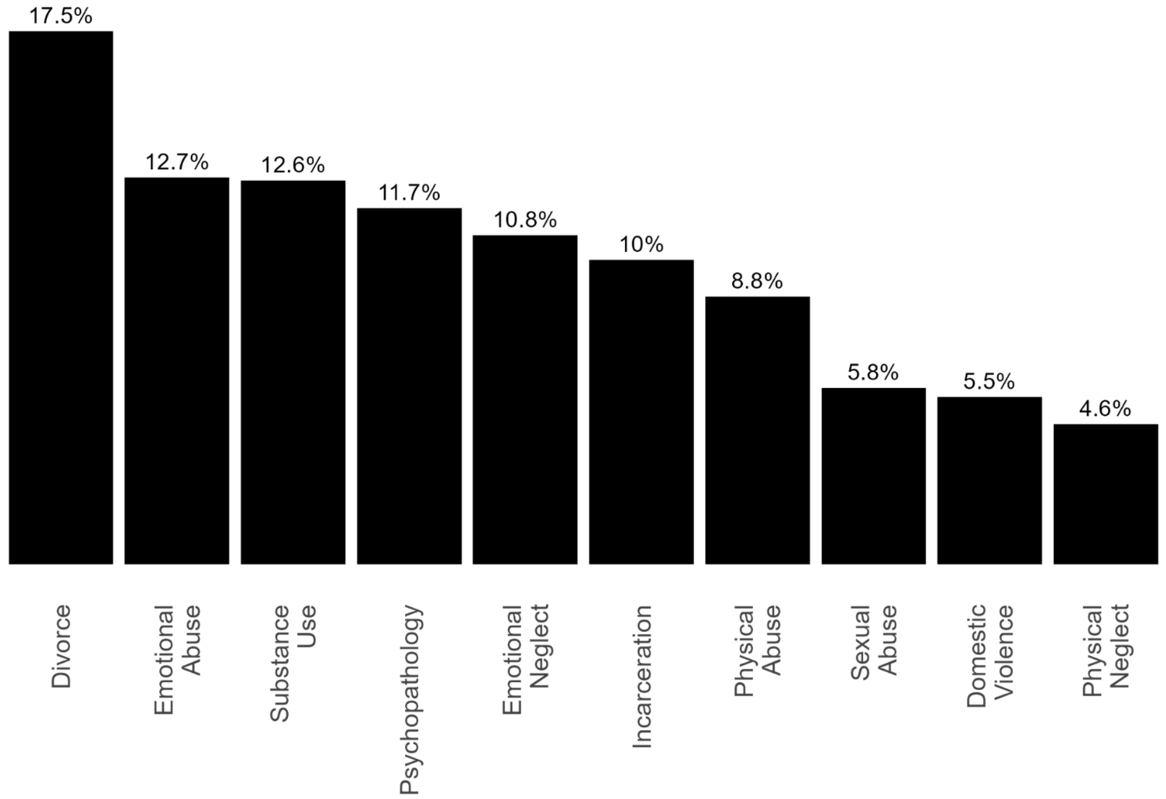

Figure S2. Proportion of maternal adverse childhood experience (ACEs) by type.

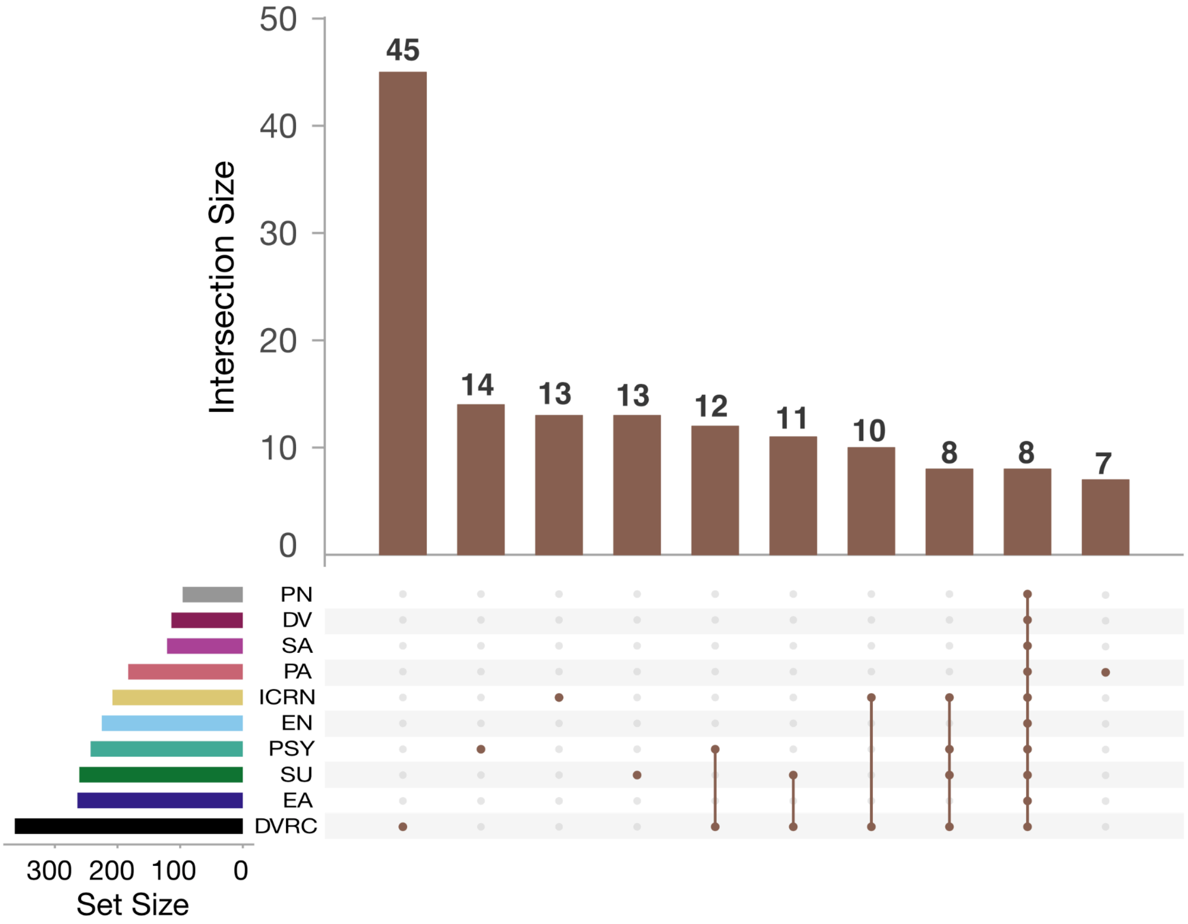

Figure S3. UpSet plot illustrating intersections among types of maternal adverse childhood experiences (ACEs). Horizontal bars show set sizes, and vertical bars show intersection sizes. Filled circles connected by lines indicate the combinations of sets contributing to each intersection.

Table S1. Correlation Matrix

	Maternal age[1]	Maternal education[2]	SES[3]	Infant's age[4]	ACEs[5]	Depression[6]	HPB
1	–	0,28*	0,25*	0,05	-0,15	-0,26*	0,05
2	–	–	0,40**	-0,01	0,00	-0,10	0,10
3	–	–	–	0,05	0,00	-0,13	0,03
4	–	–	–	–	-0,10	-0,03	0,15
5	–	–	–	–	–	0,36*	-0,11
6	–	–	–	–	–	–	-0,07

*Correlation strength: * = weak; ** = moderate. SES: Socioeconomic Status. ACEs: Adverse Childhood Experiences. HPB: Human Parenting Behavior. Superscript numbers: the line in which each variable is placed.*
